# Hypoxia-Inducible Expression of Annexin A6 Enhances the Resistance of Triple-Negative Breast Cancer Cells to EGFR and AR Antagonists

**DOI:** 10.3390/cells11193007

**Published:** 2022-09-27

**Authors:** Stephen D. Williams, Tunde M. Smith, LaMonica V. Stewart, Amos M. Sakwe

**Affiliations:** 1 Department of Biochemistry, Cancer Biology, Neuroscience, and Pharmacology, School of Graduate Studies and Research, Meharry Medical College, Nashville, TN 37208, USA; 2 School of Medicine, Meharry Medical College, Nashville, TN 37208, USA

**Keywords:** hypoxia, annexin A6, triple-negative breast cancer, EGFR, Lapatinib, androgen receptor, therapy resistance

## Abstract

Physiological changes such as hypoxia in the tumor microenvironment (TME) endow cancer cells with malignant properties, leading to tumor recurrence and rapid progression. Here, we assessed the effect of hypoxia (1% Oxygen) on the tumor suppressor Annexin A6 (AnxA6) and the response of triple-negative breast cancer (TNBC) cells to epidermal growth factor receptor (EGFR) and androgen receptor (AR) targeted therapies. We demonstrate that brief exposure of TNBC cells to hypoxia (within 24 h) is associated with down regulation of AnxA6 while > 24 h exposure cell type dependently stimulated the expression of AnxA6. Hypoxia depicted by the expression and stability of HIF-1/2α led to up regulation of the HIF target genes *SLC2A1*, *PGK1* as well as AR and the AR target genes *FABP-4* and *PPAR-γ*, but the cellular levels of AnxA6 protein decreased under prolonged hypoxia. Down regulation of AnxA6 in TNBC cells inhibited, while AnxA6 over expression enhanced the expression and cellular levels of HIF-1/2α, *SLC2A1* and *PGK1*. RNAi mediated inhibition of hypoxia induced AnxA6 expression also strongly inhibited glucose uptake and ROS production in AnxA6 expressing TNBC cells. Using a luciferase reporter assay, we confirm that short-term exposure of cells to hypoxia inhibits while prolonged exposure of cells to hypoxia enhances AnxA6 promoter activity in HEK293T cells. Compared to cells cultured under normoxia, TNBC cells were more resistant to lapatinib under hypoxic conditions, and the downregulation of AnxA6 sensitized the cells to EGFR as well as AR antagonists. These data suggest that AnxA6 is a hypoxia inducible gene and that targeting AnxA6 upregulation may be beneficial in overcoming TNBC resistance to EGFR and/or AR targeted therapies.

## 1. Introduction

Physiological changes such as hypoxia in the tumor microenvironment (TME) endow cancer cells with malignant properties, leading to tumor recurrence, rapid progression, metastasis, and drug-resistance in many solid tumor cancers, including breast cancer. These phenomena are initiated in response to hypoxia-inducible factors (HIFs) HIF-1α or HIF-2α, that sense low oxygen levels in tissues [1,2,3,4]. In triple-negative breast cancer (TNBC), overexpression of HIF-1α is associated with poor outcome in early-stage disease [5]. In a recent study, HIF-1α-specific IgG was shown to be elevated in TNBC patients and immunization against HIF-1α inhibited the growth of basal-like mammary tumors [6]. Moreover, TNBCs frequently show pathologic evidence of hypoxia including central fibrosis and necrosis [7,8], suggesting the upregulation of HIF-1α in triple-negative breast tumors. This is supported by the preferential expression of HIF-1α in peri-necrotic tumor cells in TNBC and BRCA1 mutated breast cancers [9]. Preclinical studies in mouse models have also demonstrated that loss of HIF-1α or HIF-2α expression impairs the metastasis of breast cancer cells to axillary lymph nodes [10], lungs [11,12] and bone [13,14]. Specific HIF target genes have been identified under hypoxia in breast cancer cells that promote critical steps in the metastatic process including stromal cell recruitment [15], cancer cell migration [16], invasion and intravasation [17,18,19,20,21], extravasation [14], and pre-metastatic niche formation [17,21]. Moreover, the increased expression of these HIF target genes in primary breast cancers is associated with increased patient mortality [22].

It is well established that as cancer cells rapidly proliferate, they outstrip their oxygen supply and generate a hypoxic TME. This hypoxic environment in turn, is accompanied by decreased intracellular pH (pHi) [23], which has been reported to result from attenuated oxidative phosphorylation and a shift to glycolysis [24]. Over the past decade, Annexins, a family of Ca^2+^ dependent membrane binding proteins, have been implicated in cellular processes regulated by hypoxia including proliferation, transformation, apoptosis, ion-channel transport, and energy metabolism [25,26,27,28]. Specifically, Annexin A6 (AnxA6) and Annexin A2 (AnxA2) have been demonstrated to sense changes in pHi during hypoxia and that the interaction of AnxA6 with plasma and microsomal membranes increases at acidic pH [28,29]. While these AnxA6-membrane interactions are known to mediate cell functions including vesicular transport, cell growth, and motility through cell surface receptor mediated signaling [30,31,32], it remains unclear whether AnxA6 is important in the survival of breast cancer cells during hypoxia and/or targeted therapeutic interventions.

Recent studies on AnxA6 have shown that reduced expression promotes rapid tumor growth, affecting several aspects of energy metabolism [33,34,35]. The prolonged treatment of AnxA6-low TNBC cells with EGFR-tyrosine kinase inhibitors (EGFR-TKIs) induces AnxA6 expression and accumulation of cholesterol, suggestive of a novel mechanism of resistance [36]. In addition, our recent study suggests that the expression of AnxA6 in TNBC is associated with distinct metabolic adaptations of basal-like and mesenchymal-like TNBC subsets in response to cellular stress and/or therapeutic intervention [37]. Although these studies highlight the role of AnxA6 in tumor growth and therapeutic resistance, it remains unclear whether physiological stresses such as hypoxia alter the expression status of AnxA6 in TNBC cells and consequently, metabolic adaptations and the dismal response to targeted therapeutic interventions. In this study, we hypothesize that physiological stresses such as hypoxia induce the expression of AnxA6 and that induction of AnxA6 expression underlies the dismal response of TNBC cells to EGFR and androgen receptor (AR) targeted therapies. Our data suggest that hypoxia induces the expression of AnxA6 and that the hypoxia inducible AnxA6 facilitates glucose uptake and resistance of TNBC cells to EGFR and AR antagonists.

## 2. Materials and Methods

### 2.1. Cell Culture

MDA-MB-468 (MDA-468), SUM149, HCC-70 basal-like TNBC cells, as well as SUM-159, MDA-MB-231 (MDA-231), and BT-549 mesenchymal-like TNBC cell lines were purchased from American Type Culture Collection (ATCC). All cell lines were expanded, cryopreserved at −80 °C and only early passages (<passage 5) of these cell lines were used in the experiments described in this manuscript. Prophylactic mycoplasma treatment of the cells was also routinely carried out on recovery of the cell stocks from −80 °C. MDA-468 and MDA-231 cell lines were maintained in L-15 medium (Leibovitz) containing 10% fetal bovine serum (FBS), Pen/Strep (100 units/mL penicillin and 50 units/mL streptomycin) and 0.15% NaHCO_3_. BT-549 and HCC70 cell lines were cultured in DMEM/F12 containing 10% fetal bovine serum and Pen/Strep (100 units/mL penicillin and 50 units/mL streptomycin). SUM149 and SUM159 cell lines were cultured in Ham’s F12 medium containing 10% FBS, Pen/Strep (100 units/mL penicillin and 50 units/mL streptomycin), and l-glutamine (2 mM). Cell lines were maintained at 37 °C in a humidified CO_2_ incubator and sub-cultured by trypsinization using 0.25% trypsin/0.53 mM EDTA solution (Invitrogen, Carlsbad, CA, USA). Cell lines were maintained in hypoxic conditions (1% O_2_, 94% N_2_, 5% CO_2_, 37 °C) using a Bactrox Hypoxia Chamber and glovebox (Sheldon Manufacturing, Cornelius, OR, USA). Where indicated, serum-starvation of cells was achieved by culturing the cells overnight in their respective base media containing 0.5% FBS. For treatment of cells with drugs, the cells were seeded in either 10 cm dishes or 96-well plates and allowed to attach overnight in complete medium. The medium was aspirated and replaced with fresh medium containing the vehicle control dimethyl-sulfoxide (DMSO), or the indicated concentrations of the indicated drug. Drug-containing media were replaced every 48 h.

### 2.2. Antibodies and Reagents

Antibodies against AnxA6 as well as secondary anti-mouse, anti-goat, and anti-rabbit horseradish peroxidase-conjugated antibodies were purchased from Santa Cruz Biotechnology (Dallas, TX, USA). Antibody against HIF-1α was purchased from BD Biosciences (Franklin Lakes, NJ, USA). Antibodies against HIF-2α and EGFR, were purchased from Cell Signaling Technology (Beverly, MA, USA). Antibody against β-actin (ACTB) was purchased from Sigma Aldrich (St. Louis, MO, USA). Glucose uptake assay kit (Fluorometric; ab136956) and ATP Assay Kit (Colorimetric/Fluorometric; ab83355) were purchased from Abcam (Cambridge, UK). A set of EGFR-TKIs including lapatinib-ditosylate, erlotinib, gefitinib, and canertinib were purchased from BioVision Inc. (Milpitas, CA, USA). The AR antagonists bicalutamide and enzalutamide were purchased from Sigma Aldrich (St. Louis, MO, USA). Except otherwise indicated, all other reagents were purchased from Sigma Aldrich and/or Thermo Fisher Scientific (Waltham, MA, USA).

### 2.3. Plasmid Constructs

BT-549 and MDA-468 breast cancer cell lines stably transfected with non-silencing control (NSC) or AnxA6 targeting small hairpin RNAs (shRNAs) (A6sh5), respectively, were generated and validated as previously described [38]. MDA-468 cells overexpressing flag-tagged AnxA6 were generated as previously reported [35]. We verified AnxA6 expression in all TNBC cell lines by immunoblotting.

### 2.4. Western Blotting

TNBC cells were seeded in 10 cm dishes and cultured until they were 70% confluent. The cells were harvested and washed in ice-cold PBS. Whole-cell lysates were prepared in radioimmuno-precipitation assay (RIPA) buffer (50 mM Tris-HCl, pH 7.4, 1% Nonidet P-40, 0.1% sodium 151 deoxycholate, 150 mM NaCl, 1 mM EDTA) containing protease inhibitor cocktail (Sigma) and phosphatase inhibitors (20 mM sodium fluoride, 50 mM β-glycerophosphate, and 1 mM sodium orthovanadate). Cells cultured in hypoxic conditions were scraped under hypoxic conditions using PBS incubated in hypoxic chamber overnight and rapidly washed in ice-cold PBS. Cleared cell lysates were separated in 4–12% SDS-poly acrylamide gels and then transferred to nitrocellulose membranes. The membranes were subsequently probed with mouse anti-AnxA6 antibody (1:3000 dilution), mouse anti-HIF-1α (1:500 dilution), mouse anti-HIF-2α (1:750 dilution), and rabbit anti-EGFR (1:1000 dilution). Detection of β-Actin (1:10,000) was used as the loading control. The blots were revealed by enhanced chemiluminescence (Perkin Elmer), scanned, and quantified using NIH Image J software (Version 1.53, Bethesda, MD, USA).

### 2.5. RNA Isolation and qRT-PCR

**Figure 1:**
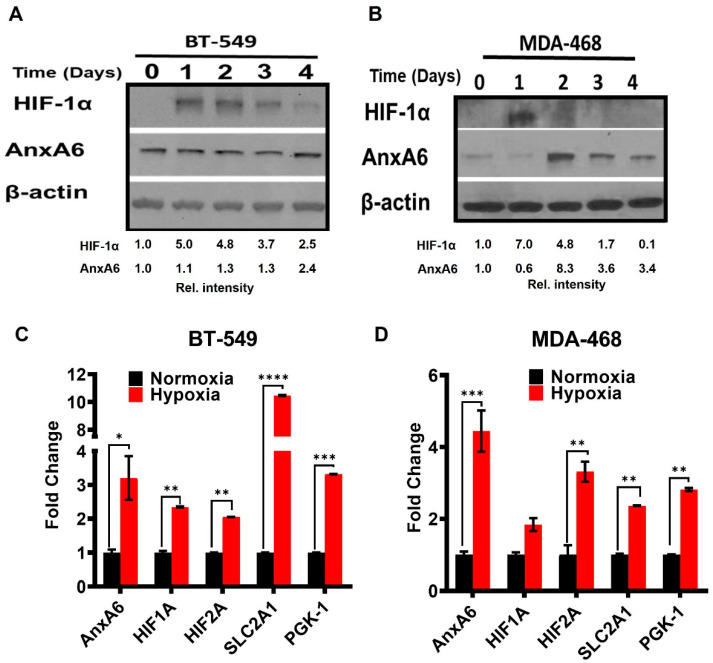
Differential Response of TNBC cells to hypoxic conditions. (**A**,**B**) Representative TNBC cell models depicting AnxA6-high mesenchymal-like (BT-549) and AnxA6-low basal-like (MDA-468) TNBC cell lines were incubated under hypoxic conditions (37 °C; 1% O_2_; 5% CO_2_; 94% N_2_) for the indicated times. Whole cell lysates were prepared and assessed by Western blotting using antibodies against the indicated proteins. Detection of β-actin was used as a loading control. (**C**,**D**) TNBC cells were cultured under hypoxic conditions for 96 h and the expression of *AnxA6*, *HIF-1/2A*, and its downstream target genes *SLCA21*- and *PGK-1* genes were assessed by RT-PCR. Statistical significance was calculated using the Mann–Whitney *U-*test in GraphPad. In parts **C**,**D**, each bar represents the mean ± SEM of duplicate analysis from three independent experiments. Asterisks (* *p* ≤ 0.05; ** *p* ≤ 0.01; *** *p* ≤ 0.001; **** *p* ≤ 0.0001) compared to normoxic values.

**Figure 2:**
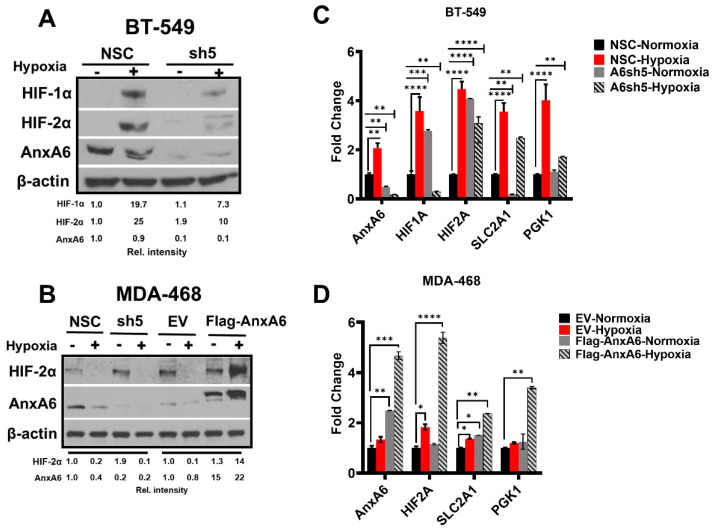
Altered Expression of AnxA6 influences HIF-1α and/or HIF-2α. (**A**) AnxA6 downregulation in the representative mesenchymal-like BT-549 TNBC cells. (**B**) Control and AnxA6 downregulation as well as empty vector and AnxA6 overexpression in the representative basal-like (MDA-468) TNBC cell line. Cells were incubated under hypoxic conditions for 96 h, and whole cell lysates were assessed by Western blotting using antibodies against HIF-1/2α and AnxA6. Detection of β-actin was used as a loading control. (**C**,**D**) Effect of AnxA6 expression status, and hypoxia on the mRNA expression of *HIF-1/2A*, and downstream target genes. In parts **C**,**D**, each bar represents the mean ± SEM of duplicate analysis from three independent experiments. Asterisks (ns *p* > 0.05; * *p* ≤ 0.05; ** *p* ≤ 0.01; *** *p* ≤ 0.001; **** *p* ≤ 0.0001). *p-*values were analyzed by Two-way ANOVA.

TNBC cell lines were grown in 10 cm dishes and cultured in complete medium under normoxic and hypoxic conditions. Total RNA was extracted using the RNeasy Mini Kit from QIAGEN (Valencia, CA). Equal amounts of RNA (1 µg) were used for cDNA synthesis using the iScript cDNA synthesis kit, according to manufacturer’s protocol (Bio-Rad Laboratories; Hercules, CA, USA). This was followed by semi-quantitative real time PCR using individual TaqMan assays and universal TaqMan gene expression master mix purchased from Thermo Fisher Scientific (Waltham, MA, USA). The ΔΔ Ct algorithm was used to calculate the relative amounts of mRNA expression of *ANXA6, HIF-1/2α, SLC2A1, PGK1, AR, FABP-4, PPARγ, 18S,* and *GAPDH*. Quantified mRNA was normalized to 18S in Figure 1C,D, Figure 2C,D, and to GAPDH for experiments in Figure 6B.

### 2.6. Luciferase Assays

**Figure 3:**
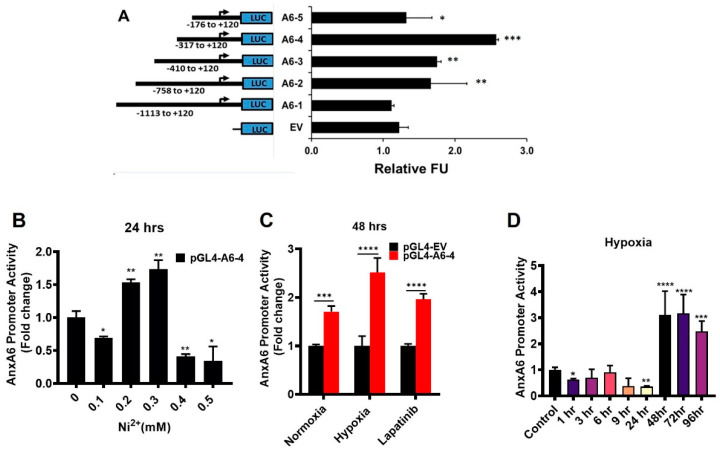
The Proximal AnxA6 promoter is responsive to Hypoxia. (**A**) The proximal promoter of AnxA6 was truncated as indicated and cloned into pGL4 basic, then used to transfect HEK293T cells and the luciferase activity was assayed following treatment of the cells with 0.3 mM Ni^2+^ for 24 h using the dual luciferase assay kit. Renilla luciferase expressing vector was used as the transfection control. (**B**) HEK293T cells transfected with pGL4-A6-4 promoter were treated with the indicated concentrations of Ni^2+^ for 24 h followed by luciferase assay. (**C**) HEK293T cells transfected with EV and pGL4-A6-4 promoter were cultured under normoxia, hypoxia, with or without lapatinib (2 µM) treatment and luciferase activity was assays as in (**A**) above. (**D**) HEK293T cells transfected with pGL4-A6-4 promoter were cultured in hypoxic conditions for up to 96 h followed by luciferase assays. All data are presented as mean ± SEM, *n = 3*. Asterisks (* *p* ≤ 0.05; ** *p* ≤ 0.01; *** *p* ≤ 0.001; **** *p* ≤ 0.0001). *p-*values were analyzed by One-way ANOVA.

The proximal AnxA6 promoter including 120 bp downstream the transcription start site (−1113 to +120) was truncated by PCR and designated A6-P1 to A6-P5 as depicted in Figure 3A. Primers included a 5′ Hind III and 3′ Xho I restriction enzymes (Thermo Fisher Scientific). The truncated promoter segments were amplified from MCF-10A genomic DNA using Phusion High Fidelity PCR master mix (New England Biolabs), digested with the restriction enzymes, and cleaned using a DNeasy plasmid purification kit (Qiagen). The fragments were cloned into Hind III/Xho I digested and purified promoter-less pGL4 luciferase reporter vector (Promega). Cloning of the AnxA6 promoter fragments were verified by restriction enzyme digestion and DNA sequencing. For luciferase assays, the AnxA6 truncated promoter constructs and a control vector expressing Renilla luciferase were co-transfected into HEK293T cells in 6-well plates using Lipofectamine 3000 (Thermo Fisher Scientific, Waltham, MA, USA). Mock-transfected and transfected HEK293T cells were cultured overnight in a humidified incubator at 37 °C. Medium was changed the following day to complete medium or medium with indicated concentrations of Ni^2+^ or Lapatinib. The effect of hypoxia on the activity of the AnxA6 promoter was assessed by incubating the transfected cells in the Bactrox hypoxia chamber for the indicated times. The cells were lysed using RIPA buffer, and luciferase activity was assessed using the dual luciferase assay kit from Promega (Madison, WI, USA), and analyzed following the manufacturer’s instructions. Renilla luciferase was used as the transfection control.

### 2.7. Cell Viability Assays

The indicated TNBC cells were seeded in 96-well plates overnight in triplicates, then treated with the indicated drugs for 72 h under normoxic and hypoxic conditions. Cell proliferation and viability were measured using the Prestoblue reagent (Invitrogen) diluted 1:10 in serum-free medium according to the manufacturer’s instructions, and the fluorescence was measured on a Synergy HT multidetection microplate reader (BioTek) at Ex/Em wavelength 535/590.

### 2.8. Glucose Uptake Assay

Glucose uptake by the indicated TNBC cell lines was determined using a glucose uptake assay kit (Colorimetric, ab136955, Abcam) as per the manufacturer’s Instructions. The indicated TNBC cell lines were seeded at a density of 2.5 × 104 cells/well in 96-well plates overnight. Cells were serum-starved for an additional 24 h and the cells in fresh complete media were incubated under normoxic or hypoxic conditions for 48 h. 2-Deoxy Glucose-6-phosphate (2-DG6P) was added to generate NADPH, which was measured at 412 nm in the Synergy HT multi-detection microplate reader (BioTek). Glucose uptake was extrapolated from a 2-DG6P standard curve according to the manufacturer’s instructions.

### 2.9. ATP Production Assay

Total ATP production was measured using an ATP Assay Kit (Abcam, ab83355). In brief, TNBC cells were incubated under normoxic or hypoxic conditions for 48 h. Culture supernatants were mixed with an ATP probe and incubated for 1 h at room temperature. Fluorescence (Ex/Em = 535/587 nm) was measured using the Synergy HT multi-detection microplate reader (BioTek).

### 2.10. ROS Generation Assay

The cellular reactive oxygen species (ROS) were quantitatively assessed using the Reactive Oxygen Species Assay Kit (Abcam #ab113851) that uses the cell permeant reagent 2′,7′-dichlorofluorescin diacetate (DCFD) following the manufacturer’s instructions (Abcam, Waltham, MA, USA). Briefly, cells were incubated under normoxic or hypoxic conditions for 48 h, then incubated with 2′,7′-dichlorodihydrofluorescein diacetate (DCFDA) probe (20 μM, 100 μL/well) for 45 min at 37 °C in the dark. The non-fluorescent DCFDA is de-acetylated by cellular esterase and then oxidized by ROS into the fluorescent 2′,7′-dichlorofluorescein (DCF), which was detected at Ex/Em 495/529 nm using the Synergy HT multi-detection microplate reader (BioTek).

### 2.11. Statistical Analysis

Data were analyzed using Mann–Whitney *U-*test. If more than two groups were compared, one-way and two-way analysis of variance (ANOVA) were performed using GraphPad Prism (San Diego, CA, USA). For all statistical analyses *p* < 0.05 was considered statistically significant.

## 3. Results

### 3.1. Differential Response of AnxA6-high vs. AnxA6-low TNBC Cells to Hypoxic Conditions

Several studies have demonstrated that hypoxia is an important component of the microenvironment of especially rapidly growing solid tumors including TNBC [39]. Although the expression status of AnxA6 influences TNBC growth [35], the relationship between AnxA6 expression and viability of TNBC cells under hypoxia has not been established. To test this, we exposed MDA-468 a basal-like TNBC cell line that expresses relatively low levels of AnxA6 and BT-549 a mesenchymal-like TNBC cell line that expresses relatively high levels of AnxA6 to hypoxia (1% O_2_) for up to 96 h and assessed the expression of AnxA6 and HIF-1α protein and mRNA. We demonstrate that HIF-1α protein expression was maximal in both cell lines following exposure to hypoxia for up to 24 h. However, following incubation of the cells under hypoxia for up to 96 h, the HIF-1α protein was rapidly lost in the AnxA6-low MDA-468 cells compared to BT-549 cells (Figure 1A,B). Longer exposure of cells to hypoxia (up to 96 h), also induced the expression of AnxA6 in a time and cell type dependent manner, with maximal expression by 48 h in MDA-468 cells and 96 h in BT-549 cells (Figure 1A,B). Similarly, in SUM149 and SUM159 cell lines that express relatively lower levels of AnxA6, hypoxic conditions were accompanied by strong induction of HIF-1α and a latent upregulation of AnxA6 (Supplementary Figure S1A,B). Given their differential response to hypoxia, our subsequent analysis was limited to the AnxA6-low MDA-468 and AnxA6-high BT-549 cell lines. By using the expression of HIF-1α and/or HIF-2α, as well as the HIF target genes solute carrier family 2 member 1 (*SLC2A1*) and Phosphoglycerate kinase 1 (*PGK1*), we next showed that incubation of cells under hypoxia for 96 h also significantly induced the expression of ANXA6 gene in both BT-549 and MDA-468 cell lines (Figure 1C,D). Interestingly, hypoxia induced expression of *SLCA21* in BT-549 cells was >4 fold that in MDA-468 cells consistent with differences in glycolytic requirements by these cell lines. Together, these data suggest that AnxA6 is upregulated during sustained exposure of TNBC cells to hypoxia and that this is accompanied by increased expression of HIF-1α target genes.

### 3.2. AnxA6 Is a Hypoxia Inducible Gene in TNBC Cells

Due to the overt changes in AnxA6 protein and mRNA expression following prolong exposure of TNBC cells to hypoxia, we next explored the effect of AnxA6 down-regulation in BT-549 and MDA-468 cell lines (Figure 2A,B) by using non-silencing control (NSC) and AnxA6 targeted small hairpin RNAs (A6sh5) transfected cells generated and validated previously [40,41]. We demonstrate that under prolong hypoxia (96 h), downregulation of AnxA6 in the AnxA6-high BT-549 cells strongly suppressed the expression of both HIF-1 and HIF-2α proteins (Figure 2A), as well as the expression of *HIF-1/2α* and *SLCA21*and *PGK1* downstream target genes. (Figure 2C). We also show that the expression of HIF-2α under this hypoxic condition was undetectable in NSC-control and AnxA6-down regulated (A6sh5) MDA-468 cells, and that overexpression of AnxA6 in this cell line [35,38], restored the stability of HIF-2α protein (Figure 2B), as well as the mRNA levels of *HIF-2A*, –*SLCA21,* and *PGK1* (Figure 2D). In these assays, HIF-1α expression was not assayed based on data in Figure 1 showing that HIF-1α protein was undetectable in the AnxA6-low MDA-468 cells and strongly attenuated in the AnxA6 high BT-549 cells following sustained (up to 96 h) exposure of the cells to hypoxia. Together with the data from Figure 1, this suggests that the expression status of AnxA6 and the inducible expression of this tumor suppressor influences the stability and/or activity of HIF-1/2α during hypoxia.

We then carried out dual luciferase reporter assays in HEK239T cells using truncated versions of the proximal AnxA6 promoter cloned upstream of firefly luciferase (LUC) as depicted in Figure 3A. For this analysis, the cells were stimulated with Ni^2+^, previously demonstrated to induce the expression of AnxA6 in AnxA6-low TNBC cells [42]. As expected, the activity of the proximal promoter of AnxA6 and specifically the fragment denoted A6-4 (−317 to +120) is stimulated in a concentration-dependent manner by Ni^2+^ (Figure 3A, B). We then confirmed that both hypoxia and the EGFR-targeting lapatinib (Figure 3C), previously shown to induce AnxA6 expression in AnxA6-low TNBC cells [36,42], also activated the −317 to +120 truncated AnxA6 promoter. Finally, this analysis showed that the activity of the −317 to +120 truncated AnxA6 promoter is time dependent with significant activity detected when the transfected cells were exposed to hypoxia for 96 h (Figure 3D). This supports data in Figure 1B suggesting that short-term exposure of TNBC cells to hypoxia consistently decreases while prolong hypoxia increases AnxA6 expression in the AnxA6-low MDA-468 TNBC cells. These data suggest that prolong treatment of AnxA6-low TNBC cells with lapatinib, calcium channel blockers and/or physiological stresses such as hypoxia leads to the activation the AnxA6 promoter and subsequent transcription of AnxA6.

### 3.3. Downregulation of AnxA6 Attenuates Glucose Uptake, ATP Production and ROS Generation in TNBC Cells under Hypoxia

**Figure 4:**
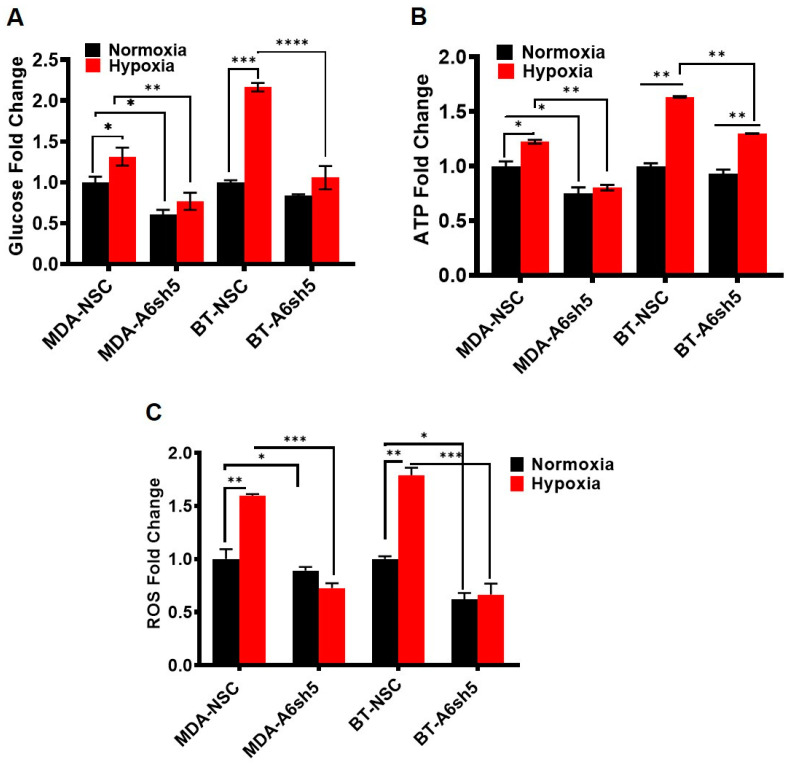
Effects of AnxA6 Downregulation on Glucose uptake, ATP production, and ROS levels during hypoxia. (**A-C**) Control AnxA6 expressing (NSC) and AnxA6 down regulated (A6sh5) MDA-468 and BT-549 TNBC cell lines were seeded in 96-well plates overnight and incubated with or without hypoxia for 48 h. Glucose uptake (**A**), ATP production (**B**), and ROS generation (**C**) were measured as described in materials and methods and normalized to normoxia values. All data are presented as mean ± SEM, *n* = 3. All data is normalized to control NSC-normoxia treatment groups for respective cell lines. Asterisks (* *p* ≤ 0.05; ** *p* ≤ 0.01; *** *p* ≤ 0.001; **** *p* ≤ 0.0001). *p-*values were analyzed by Two-way ANOVA.

Recent studies have demonstrated that AnxA6 expression status is associated with metabolic adaptation of TNBC cells [37,42] and that AnxA6-high TNBC cells exhibit the glycolytic/lipogenic phenotype while AnxA6-low cells or AnxA6 downregulation in TNBC cells led to a lipolytic phenotype [37]. Based on the hypoxia induced expression of GLUT1 and PGK1 demonstrated in Figure 1 and Figure 2, we examined whether exposure of cells to hypoxia for 48 h affects glucose uptake, as well as ATP, and reactive oxygen species (ROS) production in AnxA6 expressing (NSC) and AnxA6 down regulated (A6sh5) TNBC cells. We show that in AnxA6 expressing BT-549 cells, hypoxia leads to a significant increase (*p* = 0.0047) in glucose uptake and that this was strongly inhibited following AnxA6 down regulation (Figure 4A). Meanwhile, glucose uptake into AnxA6-low MDA-468 cells was attenuated compared to that in BT-549, and that down regulation of AnxA6 in this AnxA6-low TNBC cell line did not significantly alter the potential for glucose uptake under hypoxia or normoxia (Figure 4A). We also show a significant increase in the ATP production under hypoxia in AnxA6 expressing control BT-549 and MDA-468 cells and that this effect was attenuated following AnxA6 down regulation (Figure 4B). Similarly, ROS levels in control AnxA6 expressing MDA-468 or BT-549 cells were significantly higher under hypoxia than in normoxia and that the generation of ROS was strongly inhibited following AnxA6 down regulation in these TNBC cell types (Figure 4C). Taken together, these data suggest that AnxA6 expression status during hypoxic conditions in certain TNBC cells underlies at least in part their dependence on glycolysis.

### 3.4. Reduced AnxA6 Expression Is Associated with Increased AR Expression and Sensitivity of TNBC Cells to AR Antagonists

**Figure 5:**
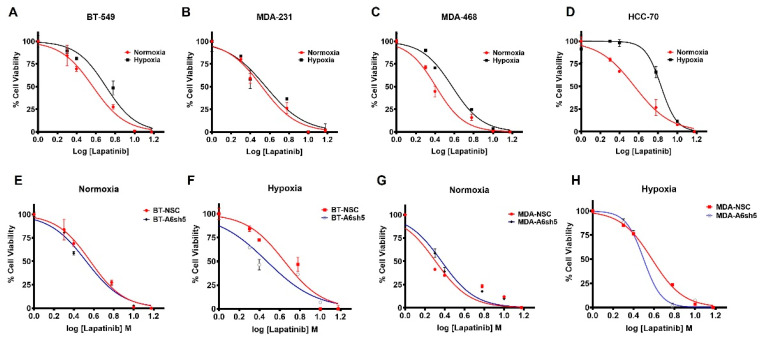
Hypoxia induced AnxA6 expression is associated with lapatinib resistance in TNBC cells Parental TNBC cells (**A,D**)**,** and control AnxA6 expressing NSC and AnxA6 down regulated (A6sh5) MDA-468 and BT-549 cells (**E,H**), were seeded at a density of 1.0 x 10^4^ cells/well in quadruplicate in 96-well plates and incubated overnight. Cells were then treated with serial dilutions of the drug ± hypoxia for a further 72 h and cell proliferation was measured using the Prestoblue cell viability reagent. Cell viability was calculated relative to untreated DMSO controls, and the IC_50_ values were calculated by non-linear regression using GraphPad prism, v.9.3. All data are presented as mean ± SEM, *n* = 4.

Although up to 70% of TNBCs express amplified levels of EGFR, targeting the receptor with TKIs and/or therapeutic monoclonal antibodies even in combination with chemotherapy has been met with dismal patient response and frequent relapse and metastasis [43,44,45]. To evaluate the effect of hypoxia induced AnxA6 on the response of TNBC cells to lapatinib treatment, we treated the AnxA6-low HCC-70 and MDA-468, as well as the AnxA6-high BT-549 and MDA-231 TNBC cell lines (Figure S2) with various concentrations of lapatinib under hypoxia or normoxia for 72 h. This analysis revealed that when cultured in hypoxic conditions, these TNBC cell lines were generally more resistant to lapatinib (Figure 5A–D, Table 1). Interestingly, HCC70 cells that express the lowest levels of AnxA6 [37] were the most resistant to lapatinib under hypoxic conditions (Figure 5D, Table 1). Meanwhile, downregulation of AnxA6 (Figure S3) sensitized BT-549 (*p* = 0.046) or MDA-468 cells (*p* = 0.038) to lapatinib especially under hypoxic conditions depicted by the significantly lower IC_50_ values for lapatinib (Figure 5E–H, Table 1). This suggests that hypoxia and/or lapatinib induced expression of AnxA6 is associated with resistance to this EGFR-TKI.

**Figure 6:**
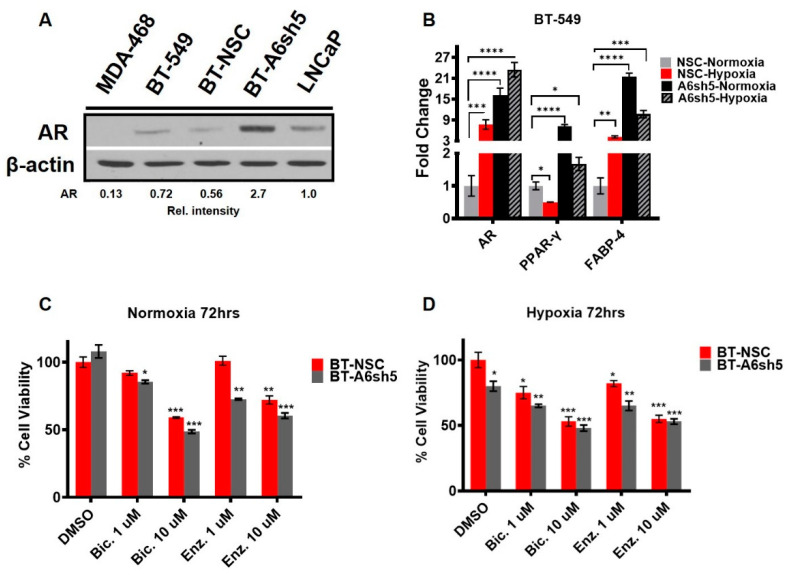
Reduced AnxA6 expression is associated with increased AR expression and sensitivity of TNBC cells to AR antagonist. (**A**) Representative Western blot showing AR expression in MDA-468, BT-549 as well as in control AnxA6 expressing and (NSC), and AnxA6 down regulated (A6sh5) BT-549 cell lines. LNCaP cells were used as the AR-positive control. Detection of β-actin was used as a loading control. (**B**) The effect of AnxA6 expression with or without hypoxia on the expression of *AR, PPARγ*, and *FABP-4* genes by qRT-PCR. (**C**, **D**) Effect of AR antagonists bicalutamide (Bic) and enzalutamide (Enz) on the viability of TNBC cells cultured under hypoxia. Cells were seeded in quadruplicate in 96 well plates, then treated with the indicated concentrations of the drugs under normoxia or hypoxia for 72 h. The viability of the cells was assessed by using the PrestoBlue cell viability assay reagent. All data are presented as mean ± SEM, *n* = 2. All data are normalized to NSC-DMSO control treatment groups for each cell line. Asterisks (* *p* ≤ 0.05; ** ≤ 0.01; *** *p* ≤ 0.001; **** *p* ≤ 0.0001) indicate significance relative to NSC-DMSO treated cells under normoxia. *p-*values were analyzed by Two-way ANOVA.

We recently showed that downregulation of AnxA6 in BT-549 cells was accompanied by increased AR signaling [35]. We first show that MDA-468 cells are AR negative and that BT-549 cells express similar levels of AR to those in the LNCaP prostate cancer cells (Figure 6A; Figure S2). Downregulation of AnxA6 in the AR positive BT-549 cells led to ~3-fold increase in the expression of AR protein (Figure 6A). Given that AR expression and/or activity is hypoxia responsive, we next assessed the effect of AnxA6 downregulation and hypoxia on the cellular levels of *AR* as well as *PPAR-γ* and *FABP-4* as representative downstream metabolic AR target genes [46,47]. This analysis revealed a 15-fold (normoxia) and 21-fold increase (hypoxia) in AR gene levels following AnxA6 down regulation in BT-549 cells (BT-A6sh5) compared to control BT-NSC cells (Figure 6B). The mRNA levels of *PPAR-γ* and *FABP-4* were correspondingly higher following AnxA6 downregulation but were attenuated following exposure of cells to low oxygen levels (Figure 6B). Treatment of control and AnxA6 downregulated BT-549 cells with the AR-antagonists, Bicalutamide and Enzalutamide, sensitized the cells to relatively low concentrations of these drugs under normoxia and hypoxia (Figure 6C,D, Table 2).Together with data in Figure 5, these data suggest that targeting AnxA6 upregulation may be beneficial in overcoming TNBC resistance to EGFR and/or AR targeted therapies.

## 4. Discussion

Triple negative breast cancer is now known to be a heterogeneous disease that is currently classified into the basal-like 1, basal-like 2, mesenchymal-like and AR positive molecular subtypes [48]. However, these molecular subtypes are far from being unique sets of TNBC due to the expression of certain markers across the subtypes, making treatment decisions even more daunting. This in part, underlies the mixed and often dismal efficacy of several targeted therapies against specific oncogenes in TNBC and other solid tumors. The use of such therapeutic options often leads to rapid development of acquired resistance and shorter relapse free survival of patients [40,41,48,49,50,51,52,53,54]. These include agents directed against the epidermal growth factor receptor as well as the less well understood AR signaling in TNBC [55,56]. The expression status of AnxA6 in TNBC cells and patient derived xenograft models has been shown to discriminate basal-like from mesenchymal-like TNBCs [42]. Several pharmacological agents have also been shown to induce the expression of AnxA6 but the effect of hypoxia, a major factor in the tumor microenvironment, on the expression of this gene and consequently, the response of TNBC cells to targeted therapies remain unclear. Our data provide evidence suggesting that AnxA6 is a hypoxia inducible gene in TNBC cells and that suppressing AnxA6 upregulation that is mediated by pharmacological agents or physiological factors may be beneficial in overcoming TNBC resistance to EGFR and/or AR targeted therapies.

Over the last decade, the tumorigenic properties of AnxA6 have been amply described in several cancer types [31,42], as well as in the resistance of TNBCs to EGFR-TKIs [35,36,38]. A recent study from our lab reported that the reciprocal expression of AnxA6 and RasGRF2 can be used to delineate rapidly growing (AnxA6-low basal-like) from highly invasive (AnxA6-high mesenchymal-like) TNBCs, which may be helpful in the prediction of the response of patients to chemotherapies and targeted therapies [42]. We also recently showed that AnxA6-expressing TNBC cells exhibited the lipogenic metabolic phenotype and dependent on glycolysis while AnxA6-low and/or AnxA6-downregulated cell lines exhibit the lipolytic metabolic phenotype and dependent on rapid fatty acid oxidation [37]. While these studies highlight the role of AnxA6 in tumor growth and therapeutic resistance, the present study revealed that physiological stresses such as hypoxia also alter AnxA6 expression in TNBC cells and consequently, metabolic adaptations and the dismal response to targeted therapeutic interventions. Although the proximal ANXA6 promoter does not appear to have a bone fide hypoxia response element by transcription factor binding site analysis, this study provides evidence supporting the notion that AnxA6 is a hypoxia-inducible gene. The inducible expression of AnxA6 as demonstrated in this study adds to the growing list of pharmacological and physiological agents that alter the expression levels of AnxA6 [42]. Most of these agents alter the expression of AnxA6 at the transcriptional level presumably by varied mechanisms.

The transcriptional activity of HIFs on over 200 downstream genes underlies the regulation of biological processes such as glucose metabolism, cell proliferation, migration, and blood vessel formation during hypoxia [57,58]. In rapidly growing tumor tissues, HIF-1α is the master regulator of the ability of hypoxic cells to shift their metabolism from oxidative phosphorylation to the less efficient glycolysis to sustain their energy demand, a phenomenon known as the Warburg Effect [59]. Our data demonstrate a distinct and/or cell-type specific modulation of the expression of HIF-1/2α proteins between the AnxA6-low basal-like MDA-468 and the AnxA6-high mesenchymal-like BT-549 cell lines when exposed to hypoxia. In particular, we show that in the AnxA6-low MDA-468 cells, the expression of HIF-1α is transient relative to that in the AnxA6-high TNBC cells. Down regulation of AnxA6 in the AnxA6-high BT-549 cells was strongly associated with reduced expression of HIFs, suggesting that the expression status of AnxA6 may be important in maintaining the levels of HIFs in TNBC cells during hypoxia. Despite this difference, upregulation of AnxA6 transcript during sustained hypoxia in both cell types was preceded by induction of HIF-1/2α. These data are consistent with studies that reported that hypoxic cells tend to consume more glucose, and this is supported by HIF-mediated induction of transporters such as *SLC2A1* that facilitate glucose import into cells and enzymes including *PGK1* that drive glycolysis [60].

Previous studies revealed that although highly stable under acute hypoxia, HIF1/2α proteins become destabilized under chronic hypoxia [61]. Our data are also consistent with this report, and it is also possible that in MDA-468 basal-like TNBC cells, the rapid loss of HIF-1α expression beyond 24 h could be regulated through O_2_-independent mechanisms reported to control HIF-1α stability and transcriptional activity including post-translational modifications such as acetylation, hydroxylation, ubiquitination, and phosphorylation [62]. The subtle differences in the changes in the expression of these genes in the EV control transfected cells may be intriguing but the trend is similar to that shown in the parental cells. The difference may be due to the smaller residual endogenous levels of AnxA6 protein in the EV cell line compared to those assessed in the parental MDA-468 cells following incubation under hypoxia for 96 h. It is possible that the difference in the expression of AnxA6 in the Flag-A6 cells could be due to the response of the CMV promoter to hypoxia as reported previously [63]. Taken together, this study reveals that hypoxia induces the expression of AnxA6 and that the expression of HIF-1/2α and HIF-dependent metabolic targets are increased in AnxA6 expressing TNBC cells.

Our data do not suggest that AnxA6 is a HIF-1/2α target gene in TNBC cells. This is supported by the distinct kinetics of AnxA6 expression versus HIF-1/2α expression in the model TNBC cell lines, and the cell type specific differences in the expression of HIF-1/2α proteins in both cell lines. It is therefore possible that the induction of AnxA6 under hypoxia is due to cellular stress. Hypoxic stress is known to contribute to tumor heterogeneity, emergence of resistance to therapeutic interventions and distinct patterns of tumor relapse and progression [64]. We have previously shown that chronic treatment of AnxA6-low TNBC cells with lapatinib also induced the expression of AnxA6 and this was associated with the development of resistance against the drug [36]. Several other compounds have been shown to induce the expression of AnxA6 including chronic treatment with non-selective calcium channel blockers [42]. Therefore, upregulation of AnxA6 under chronic hypoxia may suggest the response of TNBC cells to cellular stress and the emergence of resistant cell populations.

In our recent report, we showed that the expression status of AnxA6 underlies the metabolic plasticity and/or reprogramming of TNBC cells during metabolic stress. We demonstrated that the mesenchymal-like AnxA6-expresing TNBC cells are prone to conserve lipids and derive their energy essentially by glycolysis and therefore, depict a glycolytic/lipogenic metabolic phenotype whereas, AnxA6-low basal-like TNBC cells more readily depend on rapid uptake and oxidation of fatty acids for ATP and display the lipolytic metabolic phenotype. We also reported that lapatinib-induced AnxA6 expression in AnxA6-low TNBC cells reverted these cells from the lipolytic to the glycolytic/lipogenic phenotype, while transfection of the AnxA6-low TNBC cells with AnxA6 targeting small hairpin RNA (A6sh5) maintained their lipolytic phenotype [37]. Consistent with these observations, the present study demonstrates for the first time that AnxA6 expression is also induced by prolong hypoxia and that this is accompanied by increased expression of *SLC2A1* and *PGK1*. On the contrary, down regulation of AnxA6 in AnxA6 expressing TNBC cells inhibited the expression of these genes. This suggests that glucose uptake and glycolysis are upregulated in response to hypoxia-induced expression of AnxA6, and that metabolic adaptation and/or plasticity of TNBC cells during tumor hypoxia are AnxA6 dependent. Thus, these data support the notion that AnxA6 is a metabolic stress-response gene in TNBC, and that hypoxia induced expression of AnxA6 promotes tumor cell survival via metabolic reprogramming that favors glycolysis.

Although EGFR-targeted therapies are widely available, their efficacy in the treatment of TNBC is surprisingly poor. This may be due to the molecular heterogeneity of this breast cancer subtype but also the difficulty in effectively delineating patients who can benefit from these therapies. We recently reported a novel mechanism of acquired resistance to the EGFR targeted tyrosine kinase inhibitor lapatinib, involving drug induced upregulation of AnxA6 and accumulation of cholesterol in the lapatinib resistant cells. While this partially explains the poor efficacy of this class of drugs especially in AnxA6-low basal-like TNBCs, the role of other confounding factors including hypoxia in rapidly growing tumor cells remained unclear. Our data reveal that hypoxia mediated upregulation of AnxA6 in TNBC cells generally renders the cells resistant to EGFR as well as AR antagonists. This is consistent with the association of AnxA6 expression in tumor cells with relatively slower growth and resistance to chemotherapy. We now show that hypoxia-induced AnxA6 expression in TNBC cells also influenced the response of these cells to EGFR targeted TKIs and AR antagonists. This suggests that hypoxia induced expression of AnxA6 and potentially their dependency on glycolysis enhanced the survival of the TNBC cells following treatment with the EGFR TKIs and AR antagonists. These data are also of interest because they suggest that strategies that reduce AnxA6 expression enhance the ability of TNBC to respond to drugs that interfere with EGFR and/or AR signaling. Clinical studies suggest that only a subset of patients with AR-positive TNBC are sensitive to AR antagonists such as bicalutamide and enzalutamide [65,66]. Therefore, the altered sensitivity of TNBC cells to EGFR and AR antagonists due to hypoxia induced AnxA6 expression provides a rationale for further studies on the response of molecularly distinct TNBCs to various therapies and/or in rapidly growing and necrotic tumors in which hypoxia is a key factor in the tumor microenvironment.

## 5. Conclusions

TNBC is a spectrum of molecularly distinct and hard-to-treat breast cancers with poor prognosis. Although treatment options for TNBC are limited to cytotoxic chemotherapy, this as well as the EGFR and other targeted therapies are associated with the development of acquired resistance and rapid relapse and metastasis. Although some reports have suggested the involvement of AnxA6 in acquired resistance, it remains unclear whether physiological stresses such as hypoxia that result from chronic treatment and/or in highly aggressive tumors, alters the expression status of AnxA6 in TNBC cells and consequently, response to therapeutic interventions. Our data suggest for the first time that hypoxia induces the expression of AnxA6 and that the hypoxia inducible AnxA6 is consistent with the metabolic reprogramming and the development of resistance to EGFR and AR antagonists by TNBC cells. These data confirm that in addition to certain pharmacological agents, AnxA6 expression status is also regulated by physiological factors in the tumor microenvironment. This study suggests that targeting AnxA6 upregulation may be beneficial in overcoming the metabolic vulnerabilities and/or resistance of TNBC cells to EGFR and/or AR targeted therapies.

## Figures and Tables

**Table 1:** Effects of AnxA6 downregulation on the response of TNBC cells to lapatinib under hypoxia.

Cell Line	Normoxia IC_50_ ± SD (µM)	Hypoxia IC_50_ ± SD (µM)	*p-*Values (Normoxia vs. Hypoxia)
HCC-70	3.60 ± 0.05	6.80 ± 0.76	*p* = 0.0078
MDA-468	2.56 ± 0.08	3.78 ± 0.10	*p* = 0.03
BT-549	3.70 ± 0.37	5.05 ± 0.12	*p* = 0.01
MDA-231	3.30 ± 1.26	3.63 ± 1.90	*p* = 0.62
MDA-NSC	2.04 ± 0.28	4.07 ± 0.30 *	*p* = 0.005
MDA-A6sh5	2.33 ± 0.44	3.20 ± 0.009	*p* = 0.019
BT-NSC	3.67 ± 0.14	4.42 ± 0.37 *	*p* = 0.0418
BT-A6sh5	3.75 ± 0.41	3.84 ± 0.60	*p* = 0.865

* Denotes significantly different IC_50_ values between control AnxA6 expressing and AnxA6 down regulation in BT-549 (*p* = 0.046) and MDA-468 (*p* = 0.038) during hypoxia.

**Table 2:** Statistical significance of AnxA6 downregulation on the response to AR antagonist under hypoxia.

Treatment	Normoxia NSC vs. A6sh5	Hypoxia NSC vs. A6sh5
Bic. 1 μM	*p =* 0.01175312	*p =* 0.04217329
Bic. 10 μM	*p =* 0.00093801	*p =* 0.08194672
Enz. 1 μM	*p =* 0.00038046	*p =* 0.00621973
Enz. 10 μM	*p =* 0.0186297	*p =* 0.24763850

## Data Availability

The data presented in this study is available on request from the corresponding author.

## Supplementary Materials

The following supporting information can be downloaded at: https://www.mdpi.com/article/10.3390/cells11193007/s1, Figure S1: Differential response of SUM 149 and SUM 159 TNBC cell lines to hypoxic conditions; Figure S2: Expression of AnxA6, EGFR, and AR in TNBC and PCa cell lines; Figure S3: Expression of AnxA6 in TNBC cell lines.
