# Supplementary material for: Hypoxia-Inducible Expression of Annexin A6 Enhances the Resistance of Triple-Negative Breast Cancer Cells to EGFR and AR Antagonists
Source: Cells. 2022 Sep 27;11(19):3007. doi: 10.3390/cells11193007 (PMC9564279; doi:10.3390/cells11193007)
Supplement: Supplementary file 1 [file cells-11-03007-s001.zip › cells-1885923-supplementary.pdf]

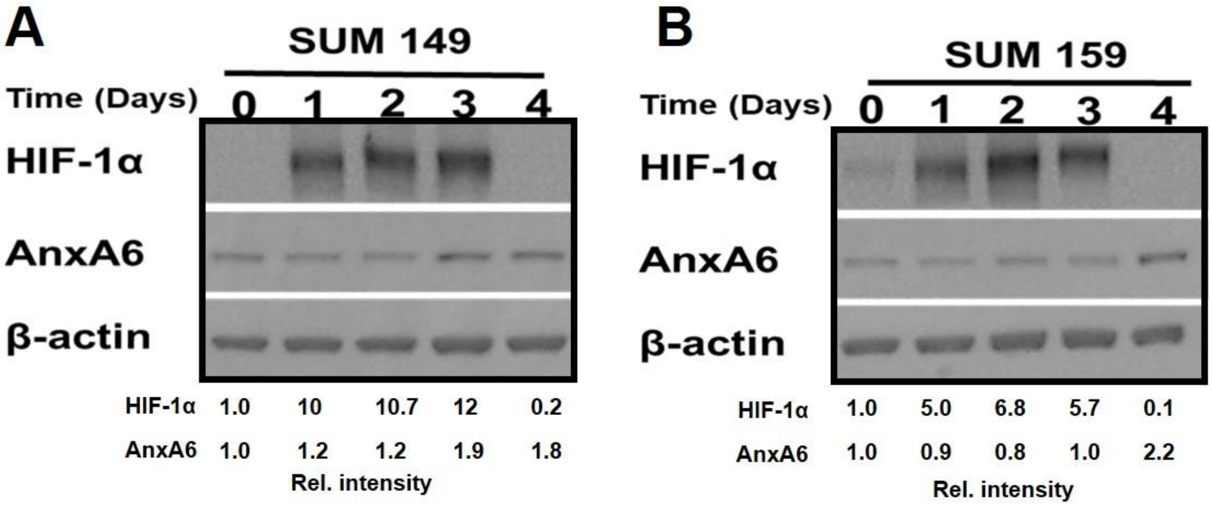

Figure S1. Differential response SUM 149 and SUM 159 TNBC cell lines to hypoxic conditions. (A-B) Representative SUM 149 and SUM 159 TNBC cell lines were incubated in acute (≤24 h) or chronic (≥24 h) hypoxic conditions (37°C; 1% O_2; 5% CO_2; 94% N_2). Whole cell lysates were prepared and assessed by western blotting using the antibodies against indicated proteins. Detection of β-actin was used as a loading control.

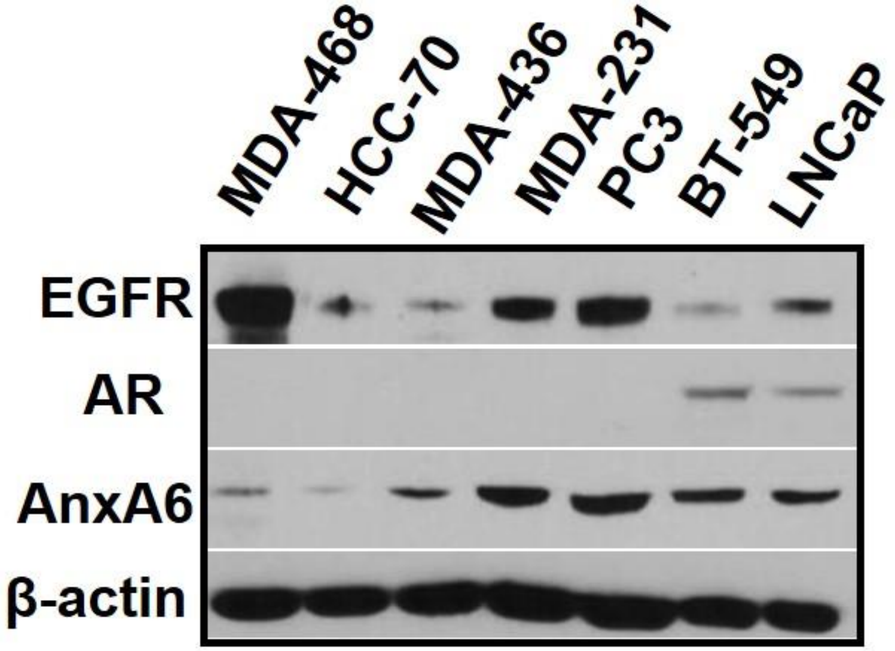

Figure S2. EGFR and AR expression status in TNBC and PCa cell lines. Panel of TNBC (MDA-468, HCC-70, MDA-436, MDA-231, and BT-549) and PCa (PC3 and LNCaP) cell lines. Whole cell lysates were

prepared and assessed by western blotting using the antibodies against EGFR, AR, and AnxA6. Detection of β-actin was used as a loading control.

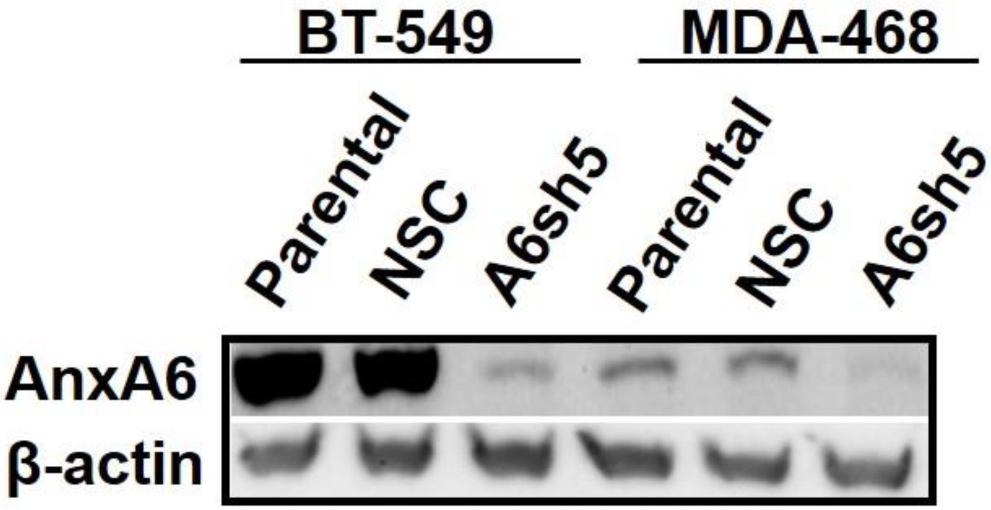

Figure S3. Expression of AnxA6 in TNBC cell lines. Representative western blot showing basal levels of AnxA6 in AnxA6-targeting NSC and A6sh5 cell lines. Whole cell lysates were prepared and assessed by western blotting using the antibodies against AnxA6. Detection of β-actin was used as a loading control.
